# P-glycoprotein transporter in drug development

**DOI:** 10.17179/excli2015-768

**Published:** 2016-02-12

**Authors:** Veda Prachayasittikul, Virapong Prachayasittikul

**Affiliations:** 1 Department of Clinical Microbiology and Applied Technology, Faculty of Medical Technology, Mahidol University, Bangkok 10700, Thailand; 2 Dental Hospital Mahidol University Faculty of Dentistry, Faculty of Dentistry, Mahidol University, Bangkok 10400, Thailand

## ⁯

Drug discovery and development is a complex and time consuming process which requires multidisciplinary expertise (Prachayasittikul et al., 2015[35]). It is true that bioactive compounds will become useless if their pharmacokinetic properties are not adequate. Pharmacokinetic properties include absorption (A), distribution (D), metabolism (M), excretion (E) and toxicity (T), or ADMET. ADMET properties influence clinical efficacy and toxicity of drugs, because they determine how much and how fast the administered drug enters the cell to reach the target site of action where it exhibits pharmacological effects, as well as control drug metabolism and elimination (van de Waterbeemd and Gifford, 2003[46]). In clinical aspect, ADMET properties determine route of administration, administered dose, and frequency of administration (van de Waterbeemd and Gifford, 2003[46]). The ADMET properties are affected by many factors including physicochemical/molecular properties of the drug (van de Waterbeemd et al., 2001[47]) and drug transporters (Lee and Kim, 2004[22]; Murakami and Takano, 2008[28]; Ueno et al., 2010[45]). Therefore, understanding the ADMET properties of candidate compounds is essential for successful drug development in terms of saving time and economic cost. In this regard, pharmacokinetic properties are important factors that need to be considered in early stages of drug development to increase the success rate and minimize financial cost (van de Waterbeemd and Gifford, 2003[46]). Computational or *in silico* approaches are effective tools for facilitating drug discovery and development (Prachayasittikul et al., 2015[35]). Computational methods are employed in many stages of drug development process, including primary ADMET screening (van de Waterbeemd and Gifford, 2003[46]). 

P-glycoprotein (Pgp) is a good example of clinical relevant drug transporter (Amin, 2013[4]; Srivalli and Lakshmi, 2012[39]; Wessler et al., 2013[52]) due to its broad-specific nature and its influence on ADMET properties of drugs (Srivalli and Lakshmi, 2012[39]). Pgp belongs to the ATP-binding cassette (ABC) superfamily (Hennessy and Spiers, 2007[16]) and is encoded by multidrug resistance (mdr) genes. Pgp expresses in many pharmacokinetic-related organs and physical barriers such as gastrointestinal (GI) tract, blood-brain-barrier (BBB), kidney, liver, endothelium and placenta (Fardel et al., 2012[11]). Pgp functions to limit cellular uptake, distribution, excretion and toxicity of a wide range of structurally unrelated hydrophobic substances, pollutants and drugs (Amin, 2013[4]) by acting as a unidirectional efflux pump, which extrudes its substrate from inside to outside of cells (Aller et al., 2009[3]). It is also recommended by the Food and Drug Administration (FDA) that a screening to ensure whether the candidate bioactive compounds are substrates of the Pgp should be conducted as early as possible during drug discovery pipeline (U.S. Food and Drug Administration, 2012[44]). Many experimental assays are available to determine interaction of the compounds against Pgp transporter (Pgp endpoint), however, discordance of experimental condition leads to conflict report of the Pgp endpoints (Polli et al., 2001[32]). Hence, classification of Pgp-interacting compounds is challenging (Wang et al., 2005[49]) and is a growing research area. Recently, many computational approaches such as quantitative structure activity relationship (Ghandadi et al., 2014[13]; Palestro et al., 2014[30]; Shen et al., 2014[38]), classification models (Adenot and Lahana, 2004[2]; Chen et al., 2011[8]; Klepsch et al., 2014[17]; Levatić et al., 2013[23]; Li et al., 2014[24]; Penzotti et al., 2002[31]; Prachayasittikul et al., 2015[34]; Wang et al., 2011[51]), molecular docking (Ghandadi et al., 2014[13]; Palestro et al., 2014[30]; Zeino et al., 2014[53]), and substructure analysis (Prachayasittikul et al., 2016[33]; Wang et al., 2011[51]; Klepsch et al., 2014[17]) have been successfully employed to provide deeper understanding about this promiscuous protein. 

The importance of Pgp is not only limited for ADMET issue, but also extended to an area of multidrug resistance (MDR) cancer (Hennessy and Spiers, 2007[16]). The linkage between Pgp overexpression and MDR cancer has been demonstrated in literatures (Abolhoda et al., 1999[1]; Thomas and Coley, 2003[42]). Increased efflux activity of the cancer cell is one of mechanisms behind drug resistance (Schinkel and Jonker, 2012[37]; Szakács et al., 2006[41]). The cancer cells derived from tissues that naturally express Pgp (i.e., kidney, colon, liver, and pancreas) have high potential to develop intrinsic drug resistance, even before exposing to anticancer agents (Sun et al., 2004[40]). Unlikely, low level of Pgp expression is found in an early diagnostic stage of cancer cells of non-Pgp expressed origin, but Pgp expression increase and the resistance is developed after treating with anticancer drugs (Fardel et al., 1996[12]; Thomas and Coley, 2003[42]). Besides exposure to anticancer agents, Pgp expression can be induced by hypoxic condition of the cancer cells (Trédan et al., 2007[43]). Pgp overexpression is found in many types (Drach et al., 1995[10]) and many stages (Krishna and Mayer, 2000[18]) of cancer cells. In addition, many clinically used anticancer agents are substrates of Pgp (Drach et al., 1995[10]). In this regard, delivery of the administered anticancer drug to target site of action is impaired thereby leading to decreased intracellular drug concentration and ineffective treatment outcome (Srivalli and Lakshmi, 2012[39]). Hence, an inhibition of Pgp function is an attractive strategy toward MDR (Szakács et al., 2006[41]). Many Pgp inhibitors (including small molecules, natural compounds, and pharmaceutical excipients (Srivalli and Lakshmi, 2012[39])) have been developed for a combination use with anticancer drugs that are substrates of the Pgp to combat resistance (Szakács et al., 2006[41]). However, the outcome remains apart from satisfaction (Szakács et al., 2006[41]).

Antimicrobial resistance is another global issue with prime concern. Efflux pump is noted to be one of the factors contributing to drug resistance of microorganisms (Rouveix, 2007[36]). Similar to MDR cancer, the MDR microorganisms express the broad-specific Pgp efflux on their components, therefore, a wide range of structurally unrelated hydrophobic antimicrobials can be extruded out of the bacterial cells (Rouveix, 2007[36]). This phenomenon limits access of the drug to target site of action and deteriorates antimicrobial effects (Rouveix, 2007[36]). Beside the search for novel antimicrobials against resistant strains, the development of efflux inhibitors (i.e., Pgp inhibitors) for co-administration with the currently used antimicrobials is considered to be an effective treatment strategy that could restore and improve effectiveness of the standard antimicrobial agents. 

It should not be overlooked that Pgp plays important roles in ADMET profiles of the administered drugs. Thus, drug-drug interaction, adverse effects and toxicities are the issues that should be concerned when many drugs are co-administered (Amin, 2013[4]; Aszalos, 2007[6]). In particular, dose adjustment and monitoring are recommended when drugs with narrow therapeutic window are co-administered with strong Pgp inhibitors (Wessler et al., 2013[52]). 

In addition to the search of novel Pgp inhibitors, modulation of Pgp expression is another strategy towards therapeutics. Abnormal Pgp expression, either increased or decreased expression, is noted to be a pathological factor of many diseases. Overexpression of Pgp in blood-brain barrier (BBB) is found in non-responsive refractory epilepsy patients and is noted to be a contributing factor of resistance against anti-epileptic drugs (Lazarowski et al., 2007[21]; Li et al., 2014[25]). Similar to cancer, Pgp expression is enhanced under the hypoxic condition, which is triggered by recurrent seizure (Li et al., 2014[25]). In this regard, suppression of Pgp expression may be an attractive treatment choice. 

Besides degenerate effects, Pgp is also noted for its protective roles. Protective role of Pgp is demonstrated in Alzheimer's disease (AD) and placenta protective mechanism. Amyloid-β is a pathologic protein of AD and its accumulation leads to neuronal damages (Hardy and Selkoe, 2002[14]). Pgp efflux pump facilitates clearance of amyloid-β from the brain and plays critical role in pathogenesis and progression of AD (Kuhnke et al., 2007[19]; Lam et al., 2001[20]). Pgp expression was found to be inversely correlated with amyloid-β deposition (Cirrito et al., 2005[9]; Hartz et al., 2010[15]; Vogelgesang et al., 2002[48]). Thus, increasing cerebrovascular Pgp expression is suggested to be an alternative therapeutic target for treatment and delay progression of AD (Brenn et al., 2014[7]). Likewise, placental Pgp efflux prevents fetus from xenobiotics, toxicants and drugs (Anger et al., 2012[5]). The protective effect of placental Pgp is correlated with level of Pgp expression. While hypoxic condition provoked increased Pgp expression (Trédan et al., 2007[43]), oxidative stress environment is noted to suppress expression and inhibit efflux function of Pgp (Li et al., 2011[27]; Wang et al., 2009[49]). Oxidative stress is one of the most common factors contributing to placental injuries and other harmful effects during pregnancy (Myatt and Cui, 2004[29]). Recent study revealed that placental Pgp expression is decreased under oxidative stress condition, and the level of Pgp expression can be restored with antioxidant agent (Li et al., 2014[26]). Hence, upregulation of placental Pgp expression may be a strategy for preventing adverse conditions and diseases in pregnancy (Li et al., 2014[26]). 

In summary, Pgp is a drug transporter of clinical importance in which many aspects of this transporter and its interacting ligands are need to be fully elucidated. Clinical relevance of Pgp and therapeutic applications of its interacting ligands render the study regarding this transporter an active research area with continual interest. The study relating to Pgp expression also could be of great benefit for understanding the unsolved problems. In clinical aspect, adjustment of dosing regimen and careful drug monitoring also take part in an effective treatment along with a maximum safety.

## Acknowledgements

This project is supported by the Office of the Higher Education Commission, Mahidol University under the National Research Universities Initiative and Annual Government Grant under Mahidol University (2556-2558 B.E.).

## Conflict of interests

The authors declare they have no conflict of interest.
